# Treatment Outcome of Regenerative Endodontic Procedures for Necrotic Immature and Mature Permanent Teeth: A Systematic Review and Meta-Analysis Based on Randomised Controlled Trials

**DOI:** 10.3290/j.ohpd.b4100877

**Published:** 2023-05-17

**Authors:** Jiahua Li, Leilei Zheng, Baraa Daraqel, Jing Liu, Yun Hu

**Affiliations:** a Postgraduate Student, Department of Stomatological Hospital of Chongqing Medical University, Chongqing, China. Chongqing Key Laboratory of Oral Diseases and Biomedical Sciences, Chongqing; Chongqing Municipal Key Laboratory of Oral Biomedical Engineering of Higher Education, Chongqing, China. First author, designed the study, collected the data, data cleaning, analysis of data, drafted the manuscript, read and approved the final manuscript.; b Professor, Department of Stomatological Hospital of Chongqing Medical University, Chongqing, China. Chongqing Key Laboratory of Oral Diseases and Biomedical Sciences, Chongqing; Chongqing Municipal Key Laboratory of Oral Biomedical Engineering of Higher Education, Chongqing, China. Obtained funding, interpretation of the results and critical revision of the manuscript for important intellectual content and approved the final version of the manuscript.; c Postgraduate Student, Department of Stomatological Hospital of Chongqing Medical University, Chongqing, China. Chongqing Key Laboratory of Oral Diseases and Biomedical Sciences, Chongqing; Chongqing Municipal Key Laboratory of Oral Biomedical Engineering of Higher Education, Chongqing, China. Data cleaning, analysis of data, interpretation of the results and critical revision of the manuscript for important intellectual content and approved the final version of the manuscript.; d Postgraduate Student, Department of Stomatological Hospital of Chongqing Medical University, Chongqing, China. Chongqing Key Laboratory of Oral Diseases and Biomedical Sciences, Chongqing; Chongqing Municipal Key Laboratory of Oral Biomedical Engineering of Higher Education, Chongqing, China. Designed the study, collected the data, read and approved the final manuscript.; e Professor, Department of Stomatological Hospital of Chongqing Medical University, Chongqing, China. Chongqing Key Laboratory of Oral Diseases and Biomedical Sciences, Chongqing; Chongqing Municipal Key Laboratory of Oral Biomedical Engineering of Higher Education, Chongqing, China. Obtained funding, designed the study, read and approved the final manuscript.

**Keywords:** dental pulp necrosis, meta-analysis, regenerative endodontics

## Abstract

**Purpose:**

To analyse whether the stage of apical development affects the effectiveness of regenerative endodontic treatment by comparing the outcomes for necrotic mature and immature permanent teeth treated with regenerative endodontic procedures.

**Materials and Methods:**

Multiple databases (PubMed, Cochrane Library, Web of Science, EMBASE and OpenGrey databases) were searched through February 17th, 2022. Inclusion criteria were randomised controlled trials that included treatment of necrotic immature or mature permanent teeth using any regenerative endodontic procedures (REPs) that aimed to achieve pulp revascularisation or regeneration. The Cochrane Risk of Bias 2.0 tool was used to assess risk of bias. The included indicators were asymptomatic sign, success, pulp sensitivity, and discolouration. The extracted data were expressed by percentage for statistical analysis. The random effect model was used to explain the results. Comprehensive Meta-Analysis Version 2 was used to perform the statistical analyses.

**Results:**

Twenty-seven RCTs were eligible for inclusion in the meta-analysis. The success rates of necrotic immature and mature permanent teeth were 95.6% (95% CI, 92.4%-97.5%; I^2^=34.9%) and 95.5% (95%CI, 87.9%-98.4%; I^2^=0%), respectively. The asymptomatic rates of necrotic immature and mature permanent teeth were 96.2% (95%CI, 93.5%-97.9%; I^2^=30.1%) and 97.0% (95%CI, 92.6%-98.8%; I^2^=0%), respectively. The treatment of immature and mature necrotic permanent teeth with REPs yields high success rates and low symptomatic rates. The incidence of positive sensitivity response for electric pulp testing in necrotic immature permanent teeth (25.2% [95% CI, 18.2%-33.8%; I^2^=0%]) was lower than that in necrotic mature permanent teeth (45.4% [95% CI, 27.2%–64.8%; I^2^=75.2%]), and this difference was statistically significant. The restoration of pulp sensitivity seems to be more evident in necrotic mature permanent teeth than in necrotic immature permanent teeth. The crown discolouration rate of immature permanent teeth was 62.5% (95% CI, 49.7%-73.8%; I^2^=76.1%). Necrotic immature permanent teeth have a considerable crown discolouration rate.

**Conclusion:**

REPs for both immature and mature necrotic permanent teeth yield high success rates and promote root development. The vitality responses seem to be more evident in necrotic mature permanent teeth than in necrotic immature permanent teeth.

Pulp necrosis occurring in immature and mature permanent teeth may require different treatment methods, considering the fact that this pathology can arrest the root development of immature teeth. Regenerative endodontic procedures (REPs) have been proposed for the treatment of immature teeth with pulp necrosis in clinical practice.^43,52^ The treatment provides a favourable environment for cell proliferation and tissue regeneration to promote root development by means of root canal disinfection, preservation of residual pulp tissue, induction of apical bleeding, an intracanal barrier and adequate restoration of the access cavity.

In 2007, Murray et al^39^ of the American Association of Endodontists proposed ‘regenerative endodontic procedures’, which could better reflect the connotation of tissue engineering in pulp treatment. The ideal histophysiological process of pulp regeneration is the formation of non-mineralised pulp tissue and mineralised dentin. However, some studies^29,57^ found that the structure of the new tissues in the root canal was disordered, showing bone-like, cementum-like, and connective tissue, containing fibroblasts and blood vessels, which was not true regeneration. Therefore, on the molecular level, the term ‘guided endodontic repair (GER)’ proposed by Diogenes et al^15^ can be used to describe this process more accurately and conservatively. In consideration of this study’s focus on clinical and radiological results, ‘regenerative endodontic procedures’ was still used to describe this procedure.

The concept of regenerative endodontics has been extended to cover necrotic mature permanent teeth, elucidating the potential of REPs to be used as substitution therapy for mature teeth with pulp necrosis. Without the concern about thickening of root canal and apical closure, REPs still have great application potential when performed in necrotic mature permanent teeth, by virtue of their being less technique sensitive and requiring less chair time.^25^ In contrast to conventional root canal treatment, REPs usually induce blood clotting to achieve biological obturation and encourage repair, rather than mechanical obturation e.g. with gutta-percha, resulting in the acquisition of immune defense mechanisms to resist reinfection. Despite the advantages of REPs, their clinical application in necrotic mature permanent teeth remains questionable. These doubts about the effect of REPs on necrotic mature permanent teeth mainly stem from differences in the apical foramen and cellular composition between mature and immature permanent teeth.^20^ Regarding the apical foramen, which is the limitation for apical pathways of stem cell migration and rush of blood, it has been suggested that the success rate of pulpal healing was significantly reduced, becoming unpredictable when the diameter of the apical foramen was less than 1 mm.^6^ However, there have been successful cases of revascularisation with an apical foramen smaller than 1.0 mm in diameter.^50^ As for cellular composition, which forms the biological basis of regenerative endodontics, there are more stem cells in immature permanent teeth than in mature permanent teeth.^44^ Despite mechanised instrumentation in mature teeth (as opposed to passive intracanal decontamination in immature teeth), Lovelace et al^35^ showed that induced bleeding could result in the accumulation of large numbers of undifferentiated stem cells into the root canal space of immature permanent teeth. Similarly, Chrepa et al^14^ showed that induced bleeding in REPs could also bring about a substantial influx of mesenchymal stem cells (MSCs) into the root canal of mature permanent teeth, indicating a biologically similar theoretical framework. It is worth emphasising that the stage of root development is a multifactorial condition that not only involves the apical foramen and cellular composition. For tooth autotransplantation, the stage of root development was found to be significantly correlated with the prognosis, as shown by the significantly increased chance of pulp healing with divergent and parallel apical roots.^28^ Similar studies on REPs are lacking.

Previous systematic reviews and meta-analyses summarised the effectiveness of REPs applied to immature or mature permanent teeth with pulp necrosis, but did not compare them with unified standards.^19,43,51^ Since the publication of those analyses, more studies have emerged, justifying a new systematic analysis of the existing evidence. Thus, we performed a systematic review and meta-analysis with the aim of comparing the clinical efficacy of REPs applied to necrotic immature and mature permanent teeth and exploring whether the stage of root development affects the effectiveness of REPs from a clinical perspective. The focused research question was defined according to the PICOS (population, intervention, comparison, outcome, study design) format: ‘As assessed by randomised controlled trials, is there any difference in the clinical efficacy of REPs for the treatment of immature and mature permanent teeth with pulp necrosis?’

## Material and Methods

### Protocol and Registration

This systematic review and meta-analysis was written following the Preferred Reporting Items for Systematic Reviews and Meta-Analyses (PRISMA) statement. The protocol was registered in the PROSPERO database (registration number: CRD42021242976).

### Search Strategy

Electronic searches were conducted in the PubMed, Cochrane Library, Web of Science, EMBASE and OpenGrey databases. The search results, limited to the English language, covered publications up to 17th February, 2022; there were no restrictions on the year of publication of the included studies. The publications mentioned in the relevant studies were also incorporated. The search terms revolved around “regenerative endodontics” and were linked together by the Booleans “AND” and “OR”. The complete search strategies are available in Table 1. The titles and abstracts of the literature obtained from the search were screened independently by two reviewers. For publications that met the requirements, the full texts were reviewed according to the inclusion and exclusion criteria. When there was a disagreement between the two reviewers, a third reviewer joined, and the disagreement was resolved by consensus.

**Table 1:** Complete search strategies

Database	Search terms used
PubMed	(((((((((((((Endodontic, Regenerative[Title/Abstract]) OR (Regenerative Endodontic[Title/Abstract])) OR (Endodontics, Regenerative[Title/Abstract])) OR (pulp revascularization[Title/Abstract])) OR (pulpal regeneration[Title/Abstract])) OR (pulp revitalization[Title/Abstract])) OR (root canal revascularization[Title/Abstract])) OR (root maturation[Title/Abstract])) OR (regenerative endodontic*[Title/Abstract])) OR (regenerative endodontic therapy[Title/Abstract])) OR (regenerative endodontic treatment*[Title/Abstract])) OR (regenerative endodontic procedure*[Title/Abstract])) OR (“Regenerative Endodontics”[Mesh])) OR (pulp regeneration[Title/Abstract])
Cochrane Library	(Regenerative Endodontic):ti,ab,kw OR (pulp revascularization):ti,ab,kw OR (pulpal regeneration):ti,ab,kw OR (pulp revitalization):ti,ab,kw OR (root canal revascularization):ti,ab,kw OR (regenerative endodontic therapy):ti,ab,kw OR (regenerative endodontic treatment):ti,ab,kw OR (regenerative endodontic procedure):ti,ab,kw OR (pulp regeneration):ti,ab,kw (Word variations have been searched)
Web of Science	(TS=(Regenerative Endodontic) OR TS=(pulp revascularization) OR TS=(pulpal regeneration) OR TS=(pulp revitalization) OR TS=(root canal revascularization) OR TS=(regenerative endodontic therapy) OR TS=(regenerative endodontic treatment) OR TS=(regenerative endodontic procedure) OR TS=(pulp regeneration)) AND TS=(teeth)
EMBASE	regenerative endodontic’:ti,ab,kw OR ‘pulp revascularization’:ab,ti OR ‘pulpal regeneration’:ti,ab,kw OR ‘pulp revitalization’:ti,ab,kw OR ‘root canal revascularization’:ti,ab,kw OR ‘regenerative endodontic therapy’:ti,ab,kw OR ‘regenerative endodontic treatment’:ti,ab,kw OR ‘regenerative endodontic procedure’:ti,ab,kw OR ‘pulp regeneration’:ti,ab,kw
OpenGrey	Regenerative Endodontics
Manual search	In references lists of included studies, relevant review articles, and relevant endodontic journals

### Eligibility and Exclusion Criteria

Eligibility and exclusion criteria were defined according to PICOS (population, intervention, comparison, outcome, study design) and language formats, as shown in Table 2.

**Table 2:** Eligibility and exclusion criteria

Items	Eligibility criteria	Exclusion criteria
Population	Permanent teeth with pulp necrosis	Primary teeth
Intervention and comparison	Treatment of necrotic immature or mature permanent teeth using any REPs that tried to achieve pulp revascularisation or regeneration regardless of the use of scaffolds, intracanal medication and stem cells	Not recounting the details of the treatment procedure
Outcome	Clinical and radiographic indicators including success rate, asymptomatic rate, pulp sensitivity rate, and/or discolouration rate	Less than 6 months of follow-up
Study design	Randomised controlled trials	In-vitro, non-randomised or animal studies
Language	Studies in English	Studies not in English

### Data Extraction

The extracted information, including general information, study procedure details and outcome data, was tabulated. The extracted information consisted of: first author(s), year of publication, journal name, research grouping, patient age, sample size, type of teeth, aetiology, irrigation protocol, intracanal disinfection medication, cell/type of scaffold, use of matrix, barrier material, follow-up duration, clinical symptoms and radiological results.

In addition, randomisation methods, data measurements, and statistical analysis methods were documented.

### Risk of Bias

All studies included in our study were randomised controlled trials. We used the Cochrane Risk of Bias 2.0 tool which supplies a framework for assessing the risk of bias in the findings regardless of the type of randomised trial.^56^ The evaluation was conducted independently and in duplicate by two reviewers. Any disagreement was resolved by discussion with a third author.

### Study Subjects and Outcomes

Immature permanent teeth were defined as teeth with open apices, and mature permanent teeth were defined as teeth with apical diameters of less than 1 mm. The asymptomatic sign was defined as the absence of any clinical symptoms (i.e. sinus tract, swelling, pain on percussion/palpation). Success was defined as the absence of any clinical symptoms with the reduction or elimination in the size of the periapical lesion. Pulp sensitivity was defined as the positive response to sensitivity testing including heat, cold and (or) electric pulp tests. Discolouration was defined as the changes in crown surface colour.

### Statistical Analysis

The Cohen kappa test was determined to assess the reliability of the two reviewers in the literature search, the data extraction and the quality evaluation. Considering heterogeneity, the random effects model was used to explain the results. Indirect comparisons were performed using pooled estimates for each outcome according to the grouping.^4,55^ Heterogeneity was denoted by I^2^. When I^2^>50%, it indicated large heterogeneity across individual studies.^21^ Comprehensive Meta-Analysis Version 2 (Biostat; Englewood, NJ, USA) was used to perform the statistical analyses. For statistical analysis of indirect comparisons, statistical significance was set at p ≤ 0.05.

### Additional Analyses

Sensitivity analysis was performed by eliminating individual studies. Each time a study was deleted, a new meta-analysis was conducted with an evaluation of whether the effect size changed. If the result after deletion was statistically significantly different from the previous result, the study would be considered to have a statistically significant impact on the total effect size.

## Results

### Study Selection

**Fig 1:**
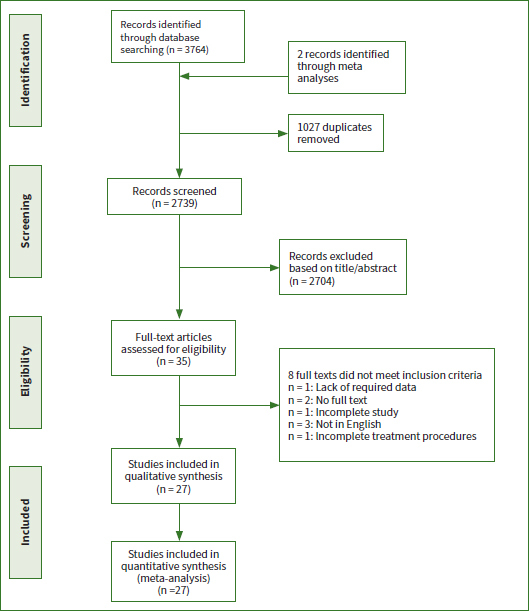
The flow diagram of the study screening and selecting procedure.

All kappa values were above 0.75. A total of 3764 articles were initially identified from the selected databases, and two studies were identified through a manual search. After removal of duplicates, 2739 articles were screened by reading their titles and abstracts. Thirty-five articles met the requirements of inclusion based on their titles and abstracts. Subsequently, the full-text assessment resulted in the exclusion of 8 articles, and the reasons for their exclusion are shown in the Fig 1. Ultimately, 27 articles^1,2,5,7,10,12,17,18,23-27,34,38,40-42,45-49,53,54,58,59^ adhered to the inclusion criteria for meta-analysis.

### Study Characteristics

In these 27 publications, 854 teeth were included, including 676 immature and 178 mature permanent teeth. The follow-up duration range of the included studies ranged from 12 to 28.25 months. The publication year ranged from 2012 to 2022. Diagnosis of pulp necrosis and outcome assessments were based on an overall analysis taking into account the aetiology, clinical symptoms and imaging data in the included studies. Only 7 studies^7,10,18,24,26,27,40^ included all outcome indicators. The aetiology of pulp necrosis was mostly trauma, caries, and developmental anomalies. The most commonly used intracanal medication was triple antibiotic paste (TAP). The most common type of capping material was mineral trioxide aggregate (MTA). Only in one study^12^ were allogenic umbilical cord mesenchymal stem cells placed into the blood clot. Grouping was accomplished through differences in the scaffold/collagen, treatment method, root canal medication, apical diameter, and capping material. Details of the included studies are summarised in Table 3.

**Table 3:** Details of the included studies

First author,year	Sample	Age	Tooth	Cause of necrosis	Irrigation protocol	Intracanal medication	Cell/scaffold	Use of matrix	Capping material	Follow-up duration
ElSheshtawy [18] 2020	31 teeth	12.66 years	AnteriorIM	Trauma/developmental anomaly	First: 5.25% NaOCl (20 ml)Second: 2.5% NaOCl (20 ml), sterile saline (20 ml), 17% EDTA (10 ml)	TAP	BC/PRP	Collagen	MTA	12 months
Jadhav [23] 2012	20 teeth	19.9 years	AnteriorIM	Not specified	2.5% NaOCl	TAP	BC/PRP	Metronidazole containing collagen	RMGIC	12 months
Ragab [45] 2019	22 teeth	9.86 years	AnteriorIM	Trauma	5% NaOCl (20 ml)+sterile saline	DAP	BC/PRF	NO	grey MTA	12 months
Rizk [46] 2020a	26 teeth	9.08 years	AnteriorIM	Trauma	First: 2% NaOCl (20 ml), 17% EDTA (20 ml)Second: sterile saline (20 ml), 17% EDTA (20 ml)	TAP	PRP/PRF	Collagen	MTA	12 months
Aly [5] 2019	26 teeth	8.96 years	AnteriorIM	Trauma /caries	First: 1.5% NaOCl (20 ml), sterile saline (20 ml)Second: 17% EDTA (20 ml)	DAP	BC	NO	Biodentine/MTA	12 months
Nagata [40] 2014	23 teeth	7-17 years	AnteriorIM	Trauma	First: 6% NaOCl (20 ml), sterile saline (10 ml), 2% CHX (10 ml)Second: 17% EDTA (3 ml), sterile saline	TAP/CHP	BC	Collagen	MTA	15 months
Jha [25] 2019	15 teeth	9-15 years	M	NR	First: 2.5% NaOClSecond: 17% EDTA	TAP	BC	NO	Cavit G	18 months
Rizk [47] 2019	26 teeth	9.08 years	Incisor IM	Trauma	First: 2% NaOCl (20 ml), 17% EDTA (20 ml)Second: sterile saline (10 ml),17% EDTA (20 ml)	TAP	BC/PRP	Collagen	MTA	12 months
Rizk [48] 2020b	26 teeth	9.08 years	Incisor IM	Trauma	First: 2% NaOCl (20 ml), 17% EDTA (20 ml)Second: sterile saline (10 ml), 17% EDTA (20 ml)	TAP	BC/PRF	Collagen	MTA	12 months
Arslan [7] 2019	28 teeth	20.58 years	M	NR	First: 1% NaOCl (5 ml/canal), 5% EDTA (5 ml/canal)Second: 1% NaOCl (5 ml), 5% EDTA (2 ml), 5 ml distilled water	TAP	BC	NO	MTA	12 months
Brizuela [12] 2020	18 teeth	27.0 years	M	NR	First: 2.5% NaOCl (20 ml)Second: 17% EDTA (20 ml)	Ca(OH) 2	BC+UC-MSC	Collagen	Biodentine	12 months
Alagl [2] 2017	32 teeth	9.47 years	IM	Trauma /caries	First: 2.5% NaOCl (20 ml), 0.12 %CHX (10 ml), sterile saline (20 ml)Second: 17% EDTA (20 ml), saline	TAP	BC/PRP	NO	MTA	12 months
Bezgin [10] 2015	21 teeth	9.95 years	IM	Trauma /caries	First: 2.5% NaOCl (20 ml), 0.12% CHX (10 ml), sterile saline (20 ml)Second: 5% EDTA (20 ml),sterile saline (20 ml)	TAP	BC/PRP	NO	MTA	18 months
El-Kateb [17] 2020	18 teeth	25.5 years	AnteriorM	Trauma/defective restoration	First: 1.5% NaOCl (20 ml)Second:1.5% NaOCl (20 ml), 17% EDTA (20 ml)	Ca(OH) 2	BC	NO	Biodentine	12 months
Jiang [27] 2017	46 teeth	10.1 years	IM	Trauma/developmental anomaly	First: 1.25% NaOCl salineSecond: 17% EDTA	Ca(OH) 2	BC	Bio-Gide/NO	MTA	15.6 months
Lin [34] 2017	80 teeth	10.5 years	IM	Trauma/developmental anomaly	First: 1.5% NaOCl (20 ml), 17% EDTA (20 ml), 0.9% saline Second: 0.9% saline, 17% EDTA (20 ml)	TAP	BC	Collagen	MTA	12 months
Nagy [41] 2014	24 teeth	10.9 years	AnteriorIM	NR	First: 2.6% NaOCl (10 ml)Second: 2.6% NaOCl (10 ml), sterile saline (10 ml)	TAP	BC	NO/Hydrogel	MTA	18 months
Santhakumar [49] 2018	40 teeth	7-12 years	AnteriorIM	NR	3% NaOCl, saline	TAP	PRF	NO	MTA	18 months
Shivashankar [54] 2017	60 teeth	6-28 years	AnteriorIM	Trauma /caries	First: 5.25% NaOClSecond: sterile saline	TAP	BC/PRF/PRP	NR	MTA	12 months
Sharma [53] 2016	16 teeth	10-25 years	AnteriorIM	Trauma	2.5% NaOCl	TAP	BC/PRF/Collagen/PLGA	Collgen	Glass ionomer	12 months
Ulusoy [58] 2019	88 teeth	8-11 years	AnteriorIM	Trauma	First: 1.25% NaOCl (20 ml)Second: sterile saline (10 ml), 17% EDTA (10 ml)	TAP	BC/PRF/PRP/PP	NO	MTA	28.25 months
Narang [42] 2015	15 teeth	<20 years	IM	Not specified	2.5% NaOCl	TAP	BC/PRP/PRF	Collagen	RMGIC	18 months
Youssef [59] 2022	20 teeth	18-40 years	AnteriorM	Not specified	First: 1.5% NaOCl (20 ml), 17% EDTA (20 ml)Second: 0.9% saline, 17% EDTA (20 ml)	Ca(OH)2	BC/PRF	NO	MTA	12 months
Abielhassan [1] 2021	45 teeth	18-50 years	AnteriorM	Not specified	1.25% NaOCl, 17% EDTA+ LASER/0.2% Nano Chitosan irrigation	NR	PRF	Collagen	Biodentine	12 months
Jayadevan [24] 2021	27 teeth	8-30 years	AnteriorIM	Trauma	First: 1.5% NaOCl, 0.9% saline, 17% EDTASecond: 0.9% saline, 17% EDTA	TAP	A-PRF/PRF	NO	Biodentine	12 months
Jiang [26] 2021	76 teeth	7-15 years	IM	Trauma/developmental anomaly	17% EDTA	NR	BC	Bio-Gide Collagen/NO	MTA	>6 months
Mittal [38] 2021	36 teeth	16-34 years	M	Not specified	First: 1.5% NaOCl,salineSecond: 1.5% NaOCl, saline, 17% EDTA	DAP	BC/PRF	Collagen/NO/Hydroxyapatite	Biodentine	12 months

CHX: chlorhexidine; CH: calcium hydroxide apexification; MTA: mineral trioxide aggregate apexification; BC: blood clot revascularisation; TAP: triple antibiotic paste; EDTA: ethylenediaminetetraacetic acid; RMGIC: resin-modified glass-ionomer cement; UC-MSC: human umbilical cord mesenchymal stem cells. NR: not reported; IM: immature; M: mature.

### Risk of Bias

All included studies were RCTs. Loss of follow-up led to missing outcome data in some of these studies.

1. Randomisation process: 22 trials were ranked as ‘low risk’, while 5 trials were ranked as ‘some concerns’.
2. Deviations from intended interventions: 12 trials were ranked as ‘high risk’, 13 trials were ranked as ‘some concerns’ and 2 trials were ranked as ‘low risk’.
3. Missing outcomes data: 5 trials were ranked as ‘high risk’, 4 trials were ranked as’some concerns’ and 18 trials were ranked as ‘low risk’.
4. Measurement of the outcome: a total of 26 trials were ranked as ‘low risk’ while one trial was ranked as ‘high risk’.
5. Selection of the reported results: 10 trials were ranked as ‘low risk’, while 17 trials were ranked as ‘some concerns’.

In the bias assessment, 15 articles were considered high risk, 10 articles were considered concerning and 2 articles were considered low risk.

**Fig 2:**
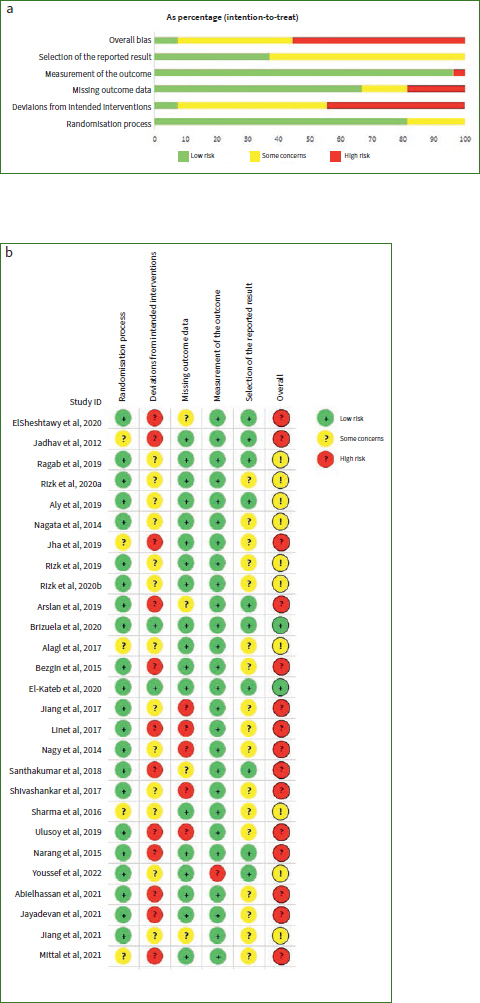
a. risk of bias graph; b. risk of bias summary.

The quality assessment details of the RCTs are shown in Fig 2.^37^

### Results of Individual Studies and Data Synthesis

The outcome measures of the included studies are shown in Table 4. The results are expressed as percentages. The primary outcome measure was the success rate.

**Table 4:** Results of the studies

First author, year	Group	Sample	Asymptomatic sign	Success	Sensibility pulp testing	Discolouration
ElSheshtawy. [18] 2020	BC	17 teeth	15 (88.2%)	NR	0 (0%)-HPT&EPT	14 (82.4%)
	PRP	13 teeth	12 (92.3%		0 (0%)-HPT&EPT	11 (84.6%)
Jadhav [23] 2012	BC	10 teeth	10 (100%)	10 (100%)	NR	NR
	PRP	10 teeth	10 (100%)	10 (100%)		
Ragab [45] 2019	BC	11 teeth	11 (100%)	11 (100%)	NR	11 (100%)
	PRF	11 teeth	11 (100%)	11 (100%)		11 (100%)
Rizk [46] 2020a	PRP	13 teeth	13 (100%)	13 (100%)	0 (0%)-CPT&HPT&EPT	NR
	PRF	12 teeth	12 (100%)	12 (100%)	0 (0%)-CPT&HPT&EPT	
Aly [5] 2019	Biodentine	13 teeth	13 (100%)	NR	NR	1 (7.69%)
	MTA	12 teeth	11 (91.67%)			7 (58.33%)
Nagata [40] 2014	TAP	12 teeth	11 (91.67%)	11 (91.67%)	0 (0%)-CPT&EPT	10 (83.3%)
	CHP	11 teeth	11 (100%)	10 (90.9%)	0 (0%)-CPT&EPT	3 (27.3%)
Jha [25] 2019	BC	15 teeth	15 (100%)	15 (100%)	NR	NR
Rizk [47] 2019	BC	13 teeth	13 (100%)	13 (100%)	0 (0%)-CPT&HPT&EPT	NR
	PRP	13 teeth	13 (100%)	13 (100%)	0 (0%)-CPT&HPT&EPT	
Rizk [48] 2020b	BC	12 teeth	12 (100%)	12 (100%)	0 (0%)-CPT&HPT&EPT	
	PRF	12 teeth	12 (100%)	12 (100%)	0 (0%)-CPT&HPT&EPT	
Arslan [7] 2019	BC	26 teeth	26 (100%)	24 (92.31%)	13 (50%)-EPT	10 (38.46%)
Brizuela [12] 2020	BC+UC-MSC	18 teeth	18 (100%)	NR	9 50%)-CPT; 5 (28%)-HPT; 6 (33%)-EPT	NR
Alagl [2] 2017	BC	15 teeth	15 (100%)	15 (100%)	13 (86.7%)-CPT&EPT	NR
	PRP	15 teeth	15 (100%)	15 (100%)	6 (40%)-CPT&EPT	
Bezgin [10] 2015	BC	10 teeth	10 (100%)	9 (90%)	2 (20%)-CPT&EPT	12 (60%)
	PRP	10 teeth	10 (100%)	10 (100%)	5 (50%)-CPT&EPT	
El-Kateb [17] 2020	X3	9 teeth	9 (100%)	9 (100%)	7 (77.8%)-CPT; 6 (66.7%)-EPT	NR
	X5	9 teeth	9 (100%)	9 (100%)	8 (88.9%)-CPT; 8 (88.9%)-EPT	
Jiang [27] 2017	Bio-Gide	21 teeth	21 (100%)	21 (100%)	7 (33%)-EPT	15 (71%)
	BC	22 teeth	22 (100%)	22 (100%)	4 (18%)-EPT	14 (64%)
Lin [34] 2017	BC	69 teeth	69 (100%)	69 (100%)	NR	30 (43.5%)
Nagy [41] 2014	BC	10 teeth	NR	9 (90%)	NR	NR
	BC+ Hydrogel	10 teeth		8 (80%)		
Santhakumar [49] 2018	PRF gel	19 teeth	18 (94.7%)	18 (94.7%)	NR	NR
	PRF membrane	19 teeth	18 (94.7%)	18 (94.7%)		
Shivashankar [54] 2017	BC	15 teeth	15 (100%)	15 (100%)	2 (13.3%)-CPT&EPT	NR
	PRF	20 teeth	20 (100%)	18 (90%)	3 (15%)-CPT&EPT	
	PRP	19 teeth	19 (100%)	19 (100%)	3 (15.8%)-CPT&EPT	
Sharma [53] 2016	BC	4 teeth	4 (100%)	4 (100%)	NR	NR
	PRF	4 teeth	4 (100%)	4 (100%)		
	BC+Collagen	4 teeth	4 (100%)	4 (100%)		
	BC+PLGA	4 teeth	4 (100%)	4 (100%)		
Ulusoy [58] 2019	BC	21 teeth	20 (95.2%)	20 (95.2%)	15 (71.4%)-CPT&EPT	NR
	PRF	17 teeth	16 (94.1%)	16 (94.1%)	11 (64.7)-CPT&EPT	
	PRP	18 teeth	18 (100%)	18 (100%)	11 (61.1%)-CPT&EPT	
	PP	17 teeth	17 (100%)	17 (100%)	13 (76.5%)-CPT&EPT	
Narang [42] 2015	BC	5 teeth	5 (100%)	5 (100%)	NR	NR
	PRF	5 teeth	5 (100%)	5 (100%)		
	PRP+Collagen	5 teeth	5 (100%)	5 (100%)		
Youssef [59] 2022	BC	10 teeth	NR	NR	2 (20%)-EPT	NR
	PRF	10 teeth			5 (50%)-EPT	
Abielhassan [1] 2021	LASER disinfection	15 teeth	15 (100%)	NR	10 (66.7 %)-EPT	NR
	Nano Chitosan irrigation	15 teeth	14 (93.3%)		11 (73.3%)-EPT	
	Conventional irrigation	15 teeth	14 (93.3%)		7 (46.7%)-EPT	
Jayadevan [24] 2021	A-PRF	14 teeth	11 (78.5%)	11 (78.5%)	0 (0%)-CPT&EPT	9 (82%)
	PRF	13 teeth	10 (77%)	10 (77%)	0 (0%)-CPT&EPT	7 (70%)
Jiang .[26] 2021	Bio-Gide Collagen	38 teeth	38 (100%)	38 (100%)	12 (31.6%)-EPT	28 (74%)
	NO Collagen	38 teeth	38 (100%)	38 (100%)	7 (18.4%)-EPT	28 (74%)
Mittal [38] 2021	BC	9 teeth	9 (100%)	9 (100%)	1 (11.1%)-CPT; 0 (0%)-HPT; 0 (0%)-EPT	NR
	PRF	9 teeth	9 (100%)	9 (100%)	6 (66.6%)-CPT; 0 (0%)-HPT; 0 (0%)-EPT	
	Collagen	9 teeth	9 (100%)	9 (100%)	4 (44.4%)-CPT; 0 (0%)-HPT; 0 (0%)-EPT	
	Hydroxyapatite	9 teeth	9 (100%)	9 (100%)	3 (33.3%)-CPT; 0 (0%)-HPT; 0 (0%)-EPT	

NR: no report; EPT: electrical pulp test; CPT: cold pulp test; HPT: heat pulp test; UC-MSCs: human umbilical cord MSCs; X3&X5: files in different types.

### Asymptomatic Rate

**Fig 3:**
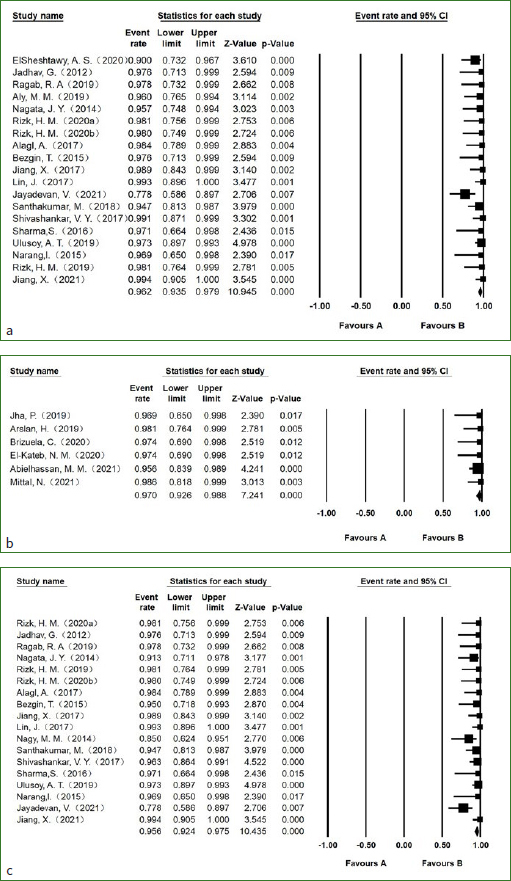

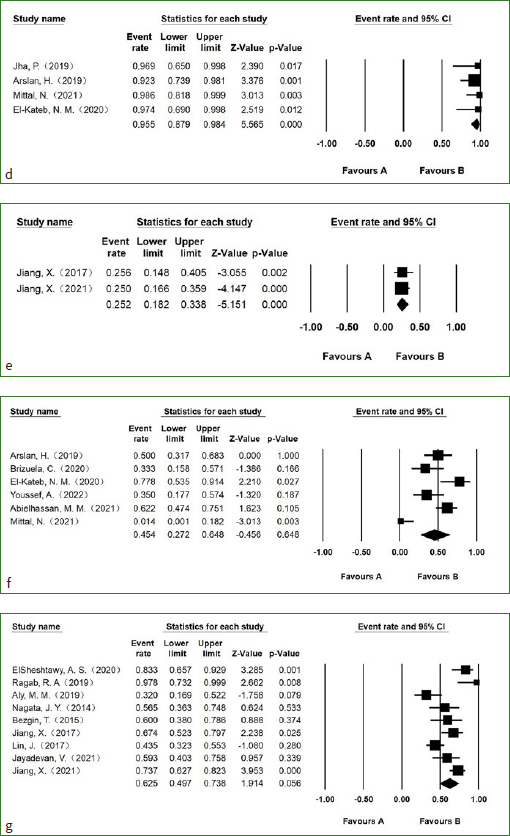
Forest plots of a. symptom rate of necrotic immature permanent teeth; b. symptom rate of necrotic mature permanent teeth; c. success rate of necrotic immature permanent teeth. Forest plots of d. success rate of necrotic mature permanent teeth; e. pulp sensitivity rate of necrotic immature permanent teeth; f. pulp sensitivity rate of necrotic mature permanent teeth; g. discolouration rate of necrotic immature permanent teeth.

For necrotic immature permanent teeth, a total of 19 studies reported this outcome indicator with an asymptomatic rate of 96.2% (95% CI, 93.5%-97.9%; I^2^=30.1%) (Fig 3a). Regarding necrotic mature permanent teeth, a total of 6 studies reported this outcome indicator with an asymptomatic rate of 97.0% (95% CI, 92.6%-98.8%; I^2^=0) (Fig 3b). There was no statistically significant difference in the rate of asymptomatic signs between necrotic immature permanent teeth and necrotic mature permanent teeth (p=0.68).

### Success Rate

The success rate of necrotic immature permanent teeth, reported in 18 studies, was 95.6% (95% CI, 92.4%-97.5%; I^2^=34.9%) (Fig 3c). The success rate of necrotic mature permanent teeth, reported in 4 studies, was 95.5% (95% CI, 87.9%-98.4%; I^2^=0) (Fig 3d). There was no statistically significant difference in the rate of success between necrotic immature permanent teeth and necrotic mature permanent teeth (p=0.97).

### Pulp Sensitivity Rate

The pooled estimate for the rate of positive response to the electrical pulp test (EPT) in 2 necrotic immature permanent teeth studies was 25.2% (95% CI, 18.2%-33.8%; I^2^=0%) (Fig 3e). The pooled estimate for the rate of positive response to EPT in 6 necrotic mature permanent teeth studies was 45.4% (95% CI, 27.2%-64.8%; I^2^=75.2%) (Fig 3f). There was a statistically significant difference in the rate of positive response to EPT between necrotic immature permanent teeth and necrotic mature permanent teeth (p=0.03).

The pooled estimate for the rate of positive response to both the cold pulp test and EPT in 9 necrotic immature permanent teeth studies was 14.5% (95% CI, 4.9%-35.6%; I^2^=88.6%). The pooled estimate for the rate of positive response to the cold pulp test in 3 necrotic mature permanent teeth studies was 57.0% (95% CI, 31.0%-79.6%; I^2^=75.7%). The pooled estimate for the rate of positive response to the heat pulp test in 2 necrotic mature permanent teeth studies was 8.6% (95% CI, 0.4%-70.4%; I^2^=79.3%).

### Discolouration Rate

Discolouration was reported in 9 studies of necrotic immature permanent teeth, with a rate of 62.5% (95% CI, 49.7%-73.8%; I^2^=76.1%) (Fig 3g). Only one study^7^ reported discolouration data for necrotic mature permanent teeth, with a discolouration rate of 38%.

### Additional Analyses

We adopted the method of eliminating single studies and found that most of the effect sizes of the two groups did not change statistically significantly. This result indicated that these study results were stable and that the sensitivity was low. Unlike most effect sizes, the effect size of positive response to the cold pulp test in the mature permanent teeth group, the heterogeneity decreased from 75.7% to 0%, and the incidence decreased from 57.0% to 42.6% when one study^17^ was removed. Considering that there were only two remaining studies after it was eliminated (which may have led to larger errors) and taking into account the variety of sensitivity tests, we kept that study.

## Discussion

The presented evidence was acquired solely from RCTs. The results of the two groups were indirectly compared,^4^ and there was a high risk of bias. The findings were consistent, except that the results for pulp sensitivity varied widely among the studies.

Since the presence of symptoms after treatment is directly related to patient satisfaction, the presence of asymptomatic signs is considered one of the outcome indicators and is discussed separately from the success rate. The results of this study showed that both necrotic immature and mature permanent teeth had high asymptomatic rates of 96.2% and 97.0%. Symptoms may be caused by incomplete crown closure and incomplete root canal disinfection. In routine REPs, MTA used as a pulp capping material is generally considered to have excellent impermeability. However, crown leakage might occur in the treated teeth after REPs, and the ability of MTA to resist bacterial penetration is questionable.^9^ All related studies have attemped to strike a balance between thorough disinfection vs toxicity to stem cells, but it is difficult to obtain a clear standard of root canal medication and the optimal clinical concentration.

In this study, success was defined as no symptoms, with reduction or elimination of the periapical lesion. Both necrotic immature and mature permanent teeth had high success rates of 95.6% and 95.5%, respectively, and there was no statistically significant difference between the two values. Based on the primary indicator, we deduced that there is no difference in the REP success rate between necrotic mature and immature permanent teeth, although REPs of necrotic mature permanent teeth are more challenging than those of necrotic immature permanent teeth. It is generally accepted that mature permanent teeth have fewer stem cells than do immature permanent teeth.^22,44^ Driesen et al^16^ reported that fully mature roots are associated with complete loss of apical papillary tissue. In addition to SCAPs, periodontal membrane stem cells and pulp stem cells around the apical region may also participate in pulp regeneration.^44^ However, the presence and quantity of SCAPs in mature permanent teeth are unknown, and an abundance of stem cells in the root area does not guarantee the success of REPs.^13^ In the studies included here, bleeding in mature permanent teeth was induced by inserting K-files into the apical area to stimulate stem cell migration. As mentioned above, the migration efficiency of stem cells may be related to the size of the apical foramen. However, the study by El-Kateb et al^17^ showed that the success rate in necrotic mature permanent teeth with REPs was not statistically significantly affected by the diameter of the apical foramen. One of the reasons may be that root canal preparation was performed in mature permanent teeth, which may provide more room for blood clots to form. Given these contradictions, more histological evidence is needed to explain the high success rate.

A positive response to the pulp sensitivity test is considered to be the clinical sign of pulp regeneration.^32^ In spite of the impossibility of evaluating the root development of mature teeth, the pulp sensitivity test could still be applied to mature teeth, for biological repair in REPs makes it possible to achieve a positive response to sensitivity testing. In this systematic analysis, the pulp sensitivity rate (EPT) of necrotic immature permanent teeth was 25.2% and that of necrotic mature permanent teeth was 45.4%; the difference between these values is statistically significant. Pulp sensitivity tests in most of the included studies were temperature tests or electrical tests, which may yield false positive symptoms; these two tests have a high misdiagnosis rate in immature permanent teeth and injured teeth.^3,33^ Studies on pulp activity have suggested that blood circulation rather than the nervous system is a more accurate determinant of pulp activity.^11^ This statement is also consistent with the post-REPs histological report^8^ that no nerve bundle was detected in the fibrous connective tissue formed in the root canal. This result suggested that REPs in necrotic mature permanent teeth may produce no less blood circulation than it does in necrotic immature permanent teeth, even without the advantage of stem cells.

Crown discolouration is a common complication in regenerative endodontic therapy. Most of the patients treated with REPs are young people, and most of the teeth involved are anterior teeth. Discolouration of the crown affects the appearance and reduces patient satisfaction. We found that necrotic immature permanent teeth had a high rate of crown discolouration (62.5%), while only one study of necrotic mature permanent teeth mentioned crown discolouration (38.5%). Minocycline and MTA used for root canal disinfection and crown obturation have been found to be associated with crown discolouration.^30,31^ Kim et al^30^ used TAP with minocycline as a root canal disinfectant during REPs and subsequently observed tooth discolouration. An in-vitro experiment^30^ verified that of the three antimicrobial agents, only minocycline caused discolouration of the crown. Kohli et al^31^ found that both gray MTA and white MTA caused crown discolouration in an in-vitro study. Bismuth oxide, a component of the MTA, has been suggested as the prime cause of staining –it reacts with the collagen in dentin matrix.^36^ Although bleaching can be used to reduce discolouration, research on the mechanism of discolouration and the materials causing it is still urgently needed.

### Limitations and Strengths

This is the first time the clinical outcomes of the two types of teeth have been compared. The present analysis confirms from a clinical point of view that the stage of root development does not affect the curative effect of REPs. All the included studies were RCTs; case reports, retrospective cohort trials and prospective cohort trials were excluded. This reduced the interference of potential unknowns to some extent. The heterogeneity of the success rate and symptom rate is low, and the heterogeneity of the pulp activity rate is high, which reduces the data reliability of this indicator. We focused on indicator assessment in pooled estimates of the frequency of each outcome and compared these estimates indirectly. Due to the lack of direct comparative studies, indirect comparison results may not accurately reflect the actual situation. The necrotic immature permanent-teeth group included 20 studies, and the necrotic mature permanent-teeth group included 7 studies. The number of necrotic mature permanent teeth is far below that of necrotic immature permanent teeth, making the distribution unbalanced. This imbalance may have led to larger discrepancies between the estimated results and the actual results.

## Conclusions

The clinical effect of REPs on necrotic mature permanent teeth may not be worse than that on necrotic immature permanent teeth, nor are the complications more numerous than those affecting necrotic immature permanent teeth. The stage of root development does not seem to affect the clinical effectiveness of REPs.

Based on the evidence of high bias and indirect comparison, the application of REPs to immature and mature necrotic permanent teeth yields high success rates and low symptomatic rates. Necrotic immature permanent teeth have a considerable crown discolouration rate. The restoration of pulp sensitivity seems to be more pronounced in necrotic mature permanent teeth than in necrotic immature permanent teeth. Further randomised clinical studies are needed to confirm the preliminary results of this review.
